# Metatranscriptomic detection of rabbit hemorrhagic disease virus 2 in karoro (southern black-backed gulls)

**DOI:** 10.1128/jvi.00781-25

**Published:** 2025-07-03

**Authors:** Stephanie J. Waller, Chris N. Niebuhr, Jessica A. Darnley, Kate McInnes, David Winter, Edward C. Holmes, Jemma L. Geoghegan

**Affiliations:** 1 Department of Microbiology and Immunology, School of Biomedical Sciences, University of Otago 626306, Dunedin, New Zealand; 2 Manaaki Whenua–Landcare Research 2243 , Lincoln, New Zealand; 3 Department of Conservation, Te Papa Atawhai 56388, Wellington, New Zealand; 4 Institute of Environmental Science and Research Ltd 8480 , Porirua, Wellington, New Zealand; 5 School of Medical Sciences, The University of Sydney 216920, Sydney, New South Wales, Australia; St. Jude Children's Research Hospital, Memphis, Tennessee, USA

**Keywords:** birds, vector, dispersal, virus, New Zealand, RHDV2

## LETTER

Rabbit hemorrhagic disease virus 2 (RHDV2; species *Lagovirus europaeus*) is a highly pathogenic lagovirus (*Caliciviridae*) responsible for a lethal disease in rabbits and hares (1). First identified in Europe in 2010 (2), RHDV2 has spread to over 35 countries (3). RHDV2 is believed to have arrived in New Zealand’s North Island in 2016 and has become established in both wild and domestic rabbit populations (3). In contrast, rabbit hemorrhagic disease virus 1 has been present in New Zealand since its illegal release as a biological control agent in 1997 (4).

**Fig 1:**
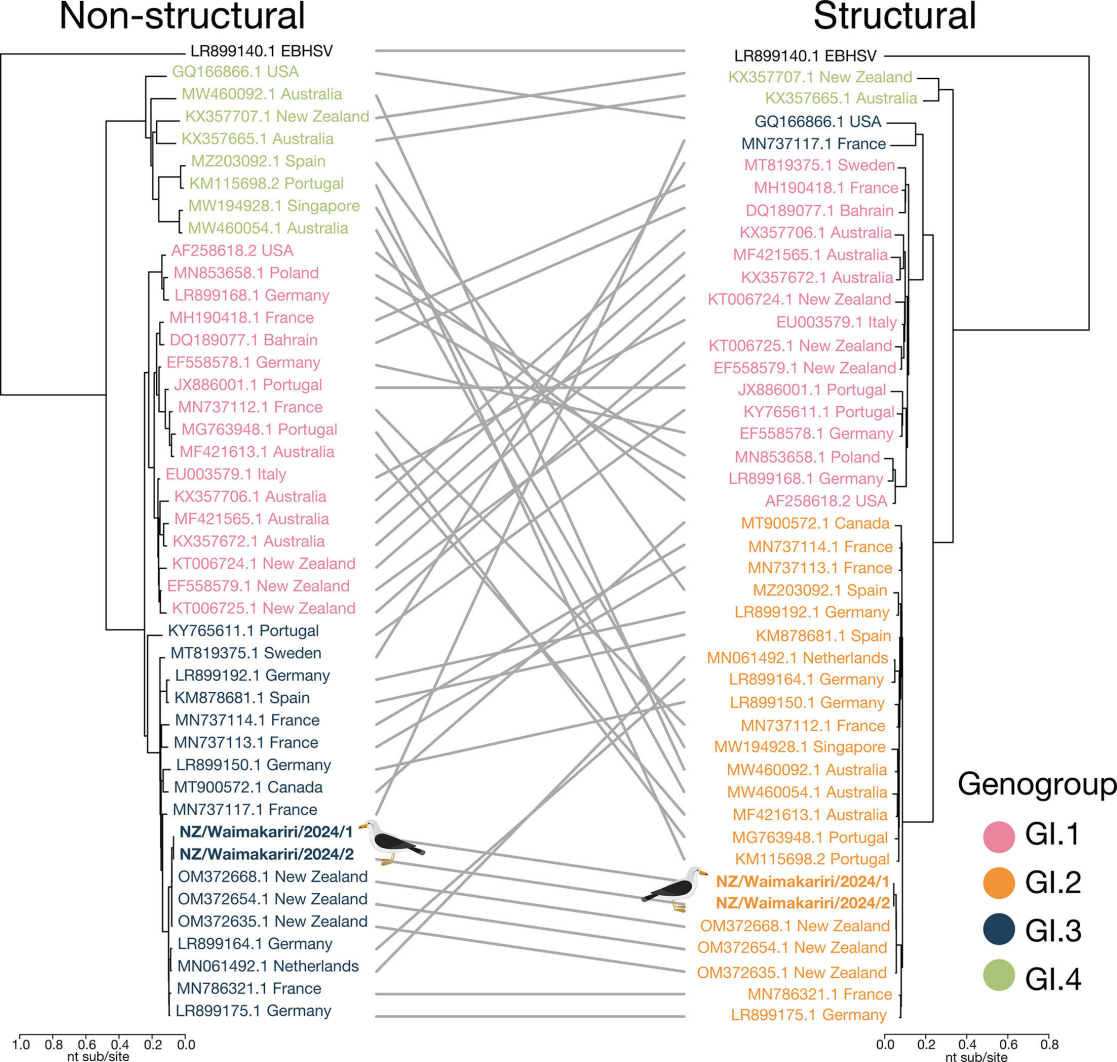
Tanglegram of New Zealand and representative global lagovirus sequences highlighting the recombinant viral genome. Representative lagovirus nucleotide sequences were obtained from NCBI/GenBank and aligned with the New Zealand RHDV2 sequences (bold) using MAFFT (12). Maximum likelihood phylogenetic trees were inferred for the non-structural (left) and structural genes (right) (nucleotide positions 28–5304 and 5305–7382, respectively [3]) using IQTREE version 1.6.12 (13), with the best-fit model as determined by ModelFinder (14). Phylogenies were rooted using European brown hare syndrome virus (EBHSV) (LR899140.1). A tanglegram was created using the ape (15) and dendextend (16) packages with the tanglegram function in RStudio version 4.3.1. Sequences are colored by genogroup, and scale bars indicate the number of nucleotide substitutions per site.

The RHDV2 strain circulating in New Zealand belongs to the GI.3P-GI.2 variant—a recombinant of the non-structural protein of a benign GI.3P lagovirus with the structural protein of GI.2 RHDV2 (3, 5) (Fig. 1). This variant has been detected in Europe (3, 5–7), North America (3, 8, 9), and China (3, 10), but notably not in Australia where RHDV2 has circulated since 2014 (3, 11). This suggests that the New Zealand incursion likely originated from outside Australia, although its precise source remains unknown (3).

Since its introduction, RHDV2 has spread within New Zealand, including at least two transmission events from the North Island to the South Island (3). The Cook Strait, which separates the islands, is approximately 23 km wide at its narrowest point. Although the exact mechanism of inter-island transmission is unknown, potential pathways include human-mediated movement, contaminated fomites, wind-assisted dispersal of fly vectors, or scavenging birds (3, 17, 18).

Through metatranscriptomic sequencing, we recovered one complete and one near-complete RHDV2 GI.3P-GI.2 genome (PV602081 and PV602082) from two RNA pools derived from oral and cloacal swabs collected in 2024 from eight southern black-backed gulls (karoro, *Larus dominicanus*) on the South Island. Oral and cloacal swabs were stored separately in DNA/RNA Shield (Zymo Research) immediately upon collection and subsequently at –80°C until total RNA was extracted using the ZymoBIOMICS MagBead RNA kit (Zymo Research). These viral sequences, with abundances of 30 (oral) and 1,159 (cloacal) reads per million, exhibited over 98% nucleotide sequence similarity in both structural and non-structural regions to the RHDV2 previously detected in European rabbits (*Oryctolagus cuniculus*) from the Otago region (South Island, New Zealand) in 2019 (3) (Fig. 1). Southern black-backed gulls are opportunistic feeders that consume a variety of terrestrial and marine organisms, as well as organic waste from farms and landfills (19). The presence of RHDV2 in these gulls is therefore likely dietary in origin, which is supported by the presence of rabbit reads in the metatranscriptomic data. In addition, RHDV2 is not believed to replicate in avian hosts.

The observation that RHDV2 is present in avian samples, albeit likely of dietary origin, makes it theoretically possible that birds act as mechanical vectors for virus transmission, perhaps through contaminated feces. This may even explain the spread of RHDV2 between New Zealand’s islands. While southern black-backed gulls are largely resident and not known for long-distance migration, there is strong evidence for their dispersal between the North and South Islands (20). The detection of complete RHDV2 genomes in these scavenger birds warrants further investigation of their potential role in viral dissemination. More broadly, these birds may influence the long-range spread of pathogens, shape viral dynamics, and present challenges for disease outbreak containment.

## Data Availability

Sequencing data generated are available in NCBI’s GenBank under accessions PV602081 and PV602082.
